# Hypotonicity modulates tetrodotoxin-sensitive sodium current in trigeminal ganglion neurons

**DOI:** 10.1186/1744-8069-7-27

**Published:** 2011-04-16

**Authors:** Lin Li, Changjin Liu, Lei Chen, Ling Chen

**Affiliations:** 1 Department of Physiology, Nanjing Medical University, Nanjing, P.R. China; 2 Department of Physiology, Tongji Medical College, Huazhong University of Science and Technology, Wuhan, P.R. China

**Keywords:** hypotonicity, tetrodotoxin-sensitive sodium current, TRPV4 receptor, intracellular signaling pathway, nociception

## Abstract

Voltage-gated sodium channels (VGSCs) play an important role in the control of membrane excitability. We previously reported that the excitability of nociceptor was increased by hypotonic stimulation. The present study tested the effect of hypotonicity on tetrodotoxin-sensitive sodium current (TTX-S current) in cultured trigeminal ganglion (TG) neurons. Our data show that after hypotonic treatment, TTX-S current was increased. In the presence of hypotonicity, voltage-dependent activation curve shifted to the hyperpolarizing direction, while the voltage-dependent inactivation curve was not affected. Transient Receptor Potential Vanilloid 4 receptor (TRPV4) activator increased TTX-S current and hypotonicity-induced increase was markedly attenuated by TRPV4 receptor blockers. We also demonstrate that inhibition of PKC attenuated hypotonicity-induced inhibition, whereas PKA system was not involved in hypotonic-response. We conclude that hypotonic stimulation enhances TTX-S current, which contributes to hypotonicity-induced nociception. TRPV4 receptor and PKC intracellular pathway are involved in the increase of TTX-S current by hypotonicity.

## Findings

**Figure 1:**
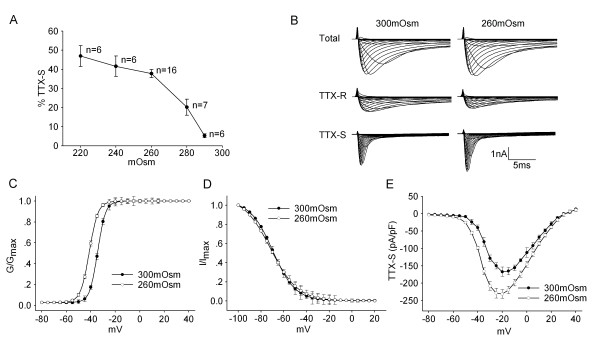
**Effect of hypotonicity on TTX-S current in TG neurons**. **A**. Plot of current densities as function of hypotonic stimuli shows that the increase of TTX-S current was conspicuous at the largest osmotic gradient. **B**. The typical recordings show that TTX-S current was increased by hypotonicity. *Upper*: total sodium current, *middle*: TTX-R current obtained during application of 300nM TTX, *below*: TTX-S current obtained by subtracting TTX-R current from total. **C**. Comparison of voltage-current relationship (I-V curve) for TTX-S current in isotonic and hypotonic solution. **D**. TTX-S current was converted to a conductance and fitted to a Boltzman function. The mid-point of activation (V_0.5_) was significantly more negative in hypotonic than in isotonic solution (-41.17 ± 1.09 mV *vs*. -34.41 ± 2.16 mV, n = 9, pared t-test, P < 0.05). However, the slope factor (*k*) was not significantly different between isotonic and hypotonic solution (3.54 ± 0.23 *vs*. 3.29 ± 0.89, n = 9, pared t-test, P > 0.05). **E**. Unlike G-V curve, the inactivation-voltage curve did not shift before and during hypotonic treatment. V_0.5 _were -69.66 ± 2.17 mV and -71.55 ± 3.21 mV (n = 10, paired t-test, P > 0.05), *k *were -9.86 ± 1.81 and -11.25 ± 1.03 (n = 10, paired t-test, P > 0.05) for 300mOsm and 260mOsm respectively.

Osmotic balance is of great significance for maintaining the internal environment homeostasis. Many pathological processes, in accompany with the changes in osmolality (such as the facial or intraoral edema which is not contained within a rigid physical restraint), are painful. Both *in vitro *and *in vivo *experiments have proved that hypotonic stimuli can induce nociception or pain-related behavior [1,2]. We previously reported that hypotonic stimulation caused an increase of action potential (AP) generation in small to medium-sized trigeminal ganglion (TG) neurons that are likely to be nociceptive in nature, resulting in the hyperexcitability of nociceptors [3]. Voltage-gated sodium channels (VGSCs), providing an inward current that underlies the upswing of an AP, contribute to the control of membrane excitability and underlie AP generation [4]. In nociceptors, VGSCs are pharmacologically separated into tetrodotoxin-sensitive (TTX-S) and tetrodotoxin-resistant (TTX-R) channels [5]. Our recent study found that TTX-R current was decreased by hypotonic stimulation [6] and this result seemingly can not explain hypotonicity-induced hyperexcitability of TG neurons. However, the modulation of VGSCs varies between laboratories and models. The selective up-regulation of TTX-S channel is detected in the pain caused by nerve injury or in inflammatory pain [7,8]. This phenomenon indicates that TTX-S channel may also play an important role in the pain sensation. Therefore, we tested the effect of hypotonic stimulation on TTX-S current in cultured small- to medium-sized TG neurons which have characteristics of nociceptors. Voltage-gated sodium current was measured first in the absence of TTX to get the total sodium current and then in the presence of TTX to obtain the TTX-R current. TTX-S current was obtained by subtracting the latter from the former. We found that TTX-S current was increased by 37.74 ± 2.12% from -160.89 ± 14.11 pA/pF to -220.18 ± 18.57 pA/pF (n = 16, paired t-test, P < 0.05) when the external solution was changed from isotonicity (300mOsm) to hypotonicity (260mOsm) (Figure 1A and 1B). Hypotonicity-induced increase was largely reversible and TTX-S current recovered to -163.75 ± 12.13 pA/pF after hypotonicity was washed out for 3 min. We also found that the voltage-dependent activation curve (G-V curve) markedly shifted to the hyperpolarizing direction in the presence of hypotonic stimulation (paired t-test, P < 0.05) (Figure 1D). Unlike the activation function, the voltage-dependent inactivation curve (inactivation-voltage curve) did not markedly shift before and during hypotonic treatment (paired t-test, P > 0.05) (Figure 1E).

**Figure 2:**
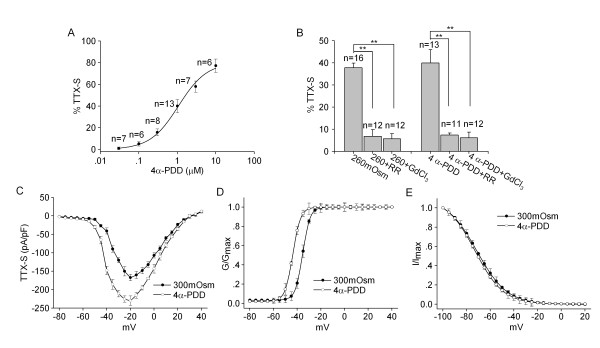
**Involvement of TRPV4 receptor in hypotonicity-induced increase of TTX-S current**. **A**. The plot shows the increase of TTX-S current by TRPV4 receptor agonist 4α-PDD at concentrations of 0.03-30 μM. The dose-response curve fits to Hill equation with EC_50 _being 1.07 μM and *n *being 1.08. **B**. The increase of TTX-S current by hypotonicity was markedly attenuated by TRPV4 receptor antagonists RR and GdCl_3_. Additionally, 4α-PDD-induced response was also significantly blocked by RR and GdCl_3_. In the presence of RR or GdCl_3_, the increase of TTX-S current by 4α-PDD (1 μM) was attenuated from 39.93 ± 6.04% to 7.36 ± 0.91% (unpaired t-test, P < 0.01) and to 6.11 ± 2.57% (unpaired t-test, P < 0.01), respectively. **C**. I-V curve shows the voltage-current relationship of TTX-S current before and during 4α-PDD (1 μM) treatment. **D**. In the presence of 4α-PDD (1 μM), G-V curve shifted to the hyperpolarizing direction, with V_0_._5 _being -35.33 ± 1.32 mV and -43.67 ± 3.72 mV (n = 8, paired t-test, P < 0.05), *k *being 3.31 ± 0.98 and 3.11 ± 0.68 (n = 8, paired t-test, P > 0.05) before and during 4α-PDD treatment, respectively. **E**. There was no significant difference in inactivation-voltage curve before and during 4α-PDD treatment. V_0_._5 _were -71.19 ± 4.15 mV and -72.72 ± 3.94 mV (n = 9, paired t-test, P > 0.05), *k *were -10.01 ± 1.05 and -10.94 ± 1.41 (n = 9, paired t-test, P > 0.05) for control and 4α-PDD group, respectively.

Transient receptor potential vanilloid subtype 4 (TRPV4) is a member of TRP super family which can be activated by multiple stimuli including hypotonicity [9-11]. As an important osmotic cellular sensor which is present in nociceptors, TRPV4 is now receiving accumulating attention concerning nociception [12]. Recent studies support an involvement of TRPV4 in anisotonicity-induced nociception [13,14]. Consistently, our recent study demonstrated that TRPV4 mediated the increase of APs number by hypotonic stimulation in TG neurons [3]. To test whether TRPV4 receptors may be involved in hypotonicity-induced modulation of TTX-S current, the agonist of TRPV4 receptor 4α-PDD was firstly used. After exposed to 4α-PDD (1 μM) for 3 min, TTX-S current was reversibly increased by 39.93 ± 6.04% from -167.46 ± 9.09 pA/pF to -230.10 ± 12.61 pA/pF (n = 13, paired t-test, P < 0.01). The increase was recoverable after 4α-PDD was washed out. 4α-PDD caused a hyperpolarizing shift in G-V curve (paired t-test, P < 0.05) (Figure 2D), but had no effect on inactivation-voltage curve (paired t-test, P > 0.05) (Figure 2E). The concentration-dependent increase of TTX-S current by 4α-PDD is shown in Figure 2A. The dose-response curve was fitted by Hill equation with EC_50 _being 1.07 μM.

To further determine whether TRPV4 receptor was involved in the effects of hypotonicity, TRPV4 receptor blockers ruthenium red (RR) and GdCl_3 _were used to determine how they would affect the increase of TTX-S current under hypotonic condition. In isotonic condition, after exposure to 10 μM RR or 100 μM GdCl_3 _for 3 min, TTX-S current was reduced from -166.07 ± 10.05 pA/pF to -135.77 ± 12.03 pA/pF (n = 8, paired t-test, P < 0.05) and from -165.89 ± 7.41 pA/pF to -140.33 ± 6.98 pA/pF (n = 9, paired t-test, P < 0.05), respectively. Upon pre-incubation with RR or GdCl_3_, the increase of TTX-S current by hypotonicity was reduced from 37.74 ± 2.12% to 6.82 ± 3.00% (unpaired t-test, P < 0.01) and to 5.67 ± 2.33% (unpaired t-test, P < 0.01), respectively. Here, it was noted that after pre-application of RR or GdCl_3 _for 3 min, the increase of TTX-S current by 4α-PDD was also significantly blocked (unpaired t-test, P < 0.01 in each case) (Figure 2B). Taken together, these data suggested that the increase of TTX-S current by hypotonic stimulation might be mediated through TRPV4 receptor.

**Figure 3:**
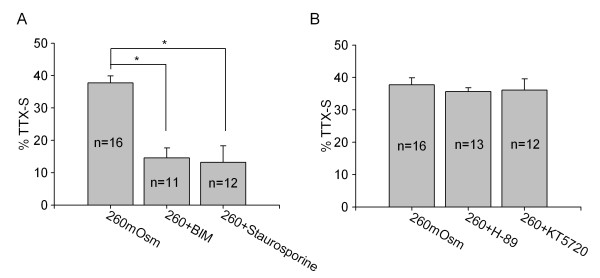
**Role of PKA and PKC system in hypotonicity-induced increase of TTX-S current**. **A**. In the presence of PKC antagonist BIM or staurosporine, hypotonicity-induced increase of TTX-S current was markedly blocked from 37.74 ± 2.12% to 14.55 ± 3.09% (unpaired t-test, P < 0.05) and to 13.17 ± 5.10% (unpaired t-test, P < 0.05), respectively. **B**. Hypotonicity-induced response was not affected by PKA antagonists and TTX-S current was increased 35.63 ± 1.17% (unpaired t-test, P > 0.05) and 36.09 ± 3.47% (unpaired t-test, P > 0.05) by hypotonicity in the presence of H-89 or KT5720, respectively.

We then tested some intracellular signaling pathways, including PKA and PKC system, to determine whether they were involved in hypotonicity-induced increase of TTX-S current. These two signaling pathways were chosen because they have been proved to be the important intracellular signal pathways modulating VGSCs [4]. We firstly compared the effect of each pathway on TTX-S current in isotonic solution. Agonist of PKA system 8-Br-cAMP (1 mM), and of PKC system phorbol-12, 13-dibutyrate (PMA, 1 μM) inhibited TTX-S current (P < 0.05 in each case). Consistently, antagonists of PKA pathway H-89 (10 μM) and KT5720 (1 μM), and of PKC pathway Bisindolylmaleimide II (BIM, 1 μM) and staurosporine (1 μM) enhanced TTX-S current (P < 0.05 in each case) (see additional file 1). Here we found that pre-treatment with PKC antagonists BIM or staurosporine markedly attenuated hypotonicity-induced increase of TTX-S current from 37.74 ± 2.12% to 14.55 ± 3.09% and to 13.17 ± 5.10% (unpaired t-test, P < 0.05 in each case), respectively. However, in the presence of H-89 or KT5720, TTX-S current was increased 35.63 ± 1.17% and 36.09 ± 3.47% by hypotonicity, respectively (unpaired t-test, P > 0.05 in each case) (Figure 3).

Nociceptors are primary afferent neurons that response to noxious stimulus and transmit the information to the central nervous system to produce pain. As VGSCs have an essential role in the biophysical properties of nociceptors, changes in both channel function and expression can lead to electrical instability of neurons, which is observed in many kinds of pain condition. Two sodium channels, a fast-inactivating TTX-S channel and a slow-inactivating TTX-R channel, predominate in small nociceptive sensory neurons. TTX-S channels are necessary for the transduction in many Aδ-fibers, whereas C-fibers responses are either wholly or partly dependent on TTX-R channels [15]. There is strong evidence that altered sodium channels play an important role in both inflammatory and neuropathic pain [7,8,16]. In the present study, a hyperpolarizing shift in the activation curve of TTX-S current was observed in the hypotonic solution, which is likely responsible for the enhancement of TTX-S current. Combined with previous study concerning TTX-R current [6], the present results support the hypothesis that sodium channel subtypes are differentially altered by hypotonic stimulation. In fact, this differential modulation of sodium channel has been observed in the pain following nerve damage, in which there is a down-regulation of TTX-R channels, but an up-regulation of TTX-S channels [7,16-19]. The enhancement of capsaicin-evoked current and trafficking of TRPV1 receptor have been noted in anisotonic stimulation [20] and more experiments need to be performed to further test whether hypotonicity had effect on TTX-S channels expression.

TRPV4 receptor is a polymodal receptor that is activated by hypotonicity, mechanical stimuli, warm heat, phorbol ester, low pH, anadamide and its LOX metabolite arachidonic acid etc. [21]. TRPV4 receptors are distributed in sensory ganglia as well as in free nerve endings and cutaneous A- and C- fiber terminals, suggesting the role in pain sensation. This idea was supported by the studies that the hypotonicity-induced increased nociceptor excitability and pain-related behavior was not present in TRPV4 knockout mice [2,3]. Besides this, activation of TRPV4 receptor promotes the release of pain-related neuropeptides (substance P and calcitonin gene-related peptide) from the central projections of primary afferents in the spinal cord [22]. In our previous report, TRPV4 receptor is involved in the modulation of ion channels, such as TTX-R current [6], voltage-gated potassium [23] and calcium channels [24]. The present study demonstrated that TRPV4 agonist 4α-PDD mimicked the effect of hypotonicity with an increase of TTX-S current and a hyperpolarizing shift of G-V curve. Additionally, the enhancement of TTX-S current by hypotonicity and 4α-PDD was markedly attenuated by TRPV4 antagonist RR and GdCl_3_. These results indicated that TRPV4 receptor was likely responsible for the modulation of TTX-S current by hypotonicity. Sodium currents are regulated by multiple signal transduction cascades. In the present study, we examined the contribution of PKA and PKC signaling pathways to the increase of TTX-S current by hypotonic stimulation. By pre-incubating the antagonists of protein kinases, we found that hypotonicity-induced increase of TTX-S current was markedly blocked by PKC antagonists but unaffected by antagonism of PKA system, which implied that PKC system was selectively involved in the hypotonic-response (Figure 3). Here, it was noted that hypotonicity-induced increase of TTX-S current was not reserved by PKC antagonists completely, indicating that other factors might contribute to the modulation. Recent study reports that Nav1.7 TTX-S channel can be modulated by ERK1/2 mitogen-activated protein kinase which is an important intracellular signaling pathway regulating multiple voltage-gated ion channels in dorsal root ganglion neurons [25]. Whether ERK1/2 pathway was involved in the increase of TTX-S current by hypotonicity needs to be proved in the future study.

In small primary sensor neurons, both TTX-S and TTX-R sodium current are important for generation of action potentials. TTX-S current which has much faster activation and inactivation kinetics than TTX-R current activates during the initial depolarization of AP, rapidly declines before the action potential reaches its peak value and remains near zero during the repolarization. On the other hand, TTX-R current does not inactivation completely during the AP and carries the majority of inward current following during the shoulder [26]. Therefore, sodium channels, which were previously thought to be simply responsible for the rising phase of an action potential, have been shown to play multiple roles in the generation of AP. Besides TTX-R current, high voltage-gated calcium current (*I*_HVA_) [24], another important contribution to the AP shoulder [26], was also inhibited by hypotonic stimulation, while voltage-gated potassium current was enhanced by hypotonicity. The above modulation would accelerate the repolarization of AP and affect the shape of the shoulder. The present results demonstrated that TTX-S current was increased by hypotonicity, which would facilitate the depolarization during an AP. In addition, the decrease of calcium influx makes it have less chance to activate calcium-activated potassium current which plays an important role in reducing the repetitive activity [27]. Therefore, hypotonicity-induced changes in the excitability and firing pattern of TG neurons may be mediated through the modulation of multiple ion channels on the cell membrane. Our present framework provides the possibility of pharmacologically targeting specific channel subtypes in treating hypotonicity-induced nociception.

## Methods and materials

### Cell culture

TG neurons from male Sprague-Dawley rats (180-200 g) were cultured as described previously [6]. Briefly, trigeminal ganglia were dissected aseptically and washed in cold (4°C) modified Hank's Balanced Salt solution (mHBSS). The ganglia were incubated in mHBSS at 37°C for 20-40 min with 0.1% collagenase (Type XI-S), triturated with a fire-polished glass pipette and finally incubated at 37°C for 10 min with 10 μg/ml DNase I (Type IV) in F-12 medium (Life Technologies, Gaithersburg, MD). Then they were centrifuged for 3 times at 5 × 1500 rpm/min and cultured in F-12 supplemented with 10% fetal bovine serum at 37°C for 24 h in a water saturated atmosphere with 5% CO_2_. The cell diameter (μm) was measured with a calibrated eyepiece under phase contrast illumination. Care of animals conformed to standards established by the National Institutes of Health. All animal protocols were approved by the Nanjing Medical University Animal Care and Use Committee. All efforts were made to minimize animal suffering and to reduce the number of animals used.

### Patch clamp recording

All experiments were carried out at room temperature (22-23°C). Whole-cell patch clamp recordings were obtained using an Axopatch-200B patch clamp amplifier (Axon Instruments, Foster City, CA) and the output was digitized with a Digidata 1322A converter (Axon Instruments). The sampling rate was 10 kHz and filtered at 5 kHz. The capacitance and series resistance were compensated ≥90%. Data obtained from neurons in which uncompensated series resistance resulted in voltage-clamp errors > 5 mV were not taken in further analysis. Liquid junction potentials were compensated before patching. When the osmolality of external solutions was changed from isotonicity to hypotonicity, measurements of the changes in liquid junction potentials were less than 2 mV and were not corrected. The glass pipettes (No. 64-0817(G85150T-3), Warner Instruments Inc., Hamden, CT, USA) with resistance of 1-3 MW when filled with pipette solution were used. In voltage-clamp experiment, the holding potential was -80 mV. The G-V curve of sodium current was measured by 20 ms depolarizing pulses from -80 to +40 mV stepping by 5 mV with interval of 2s. The inactivation-voltage curve was obtained by double pulses: precondition pulses (20 ms) were from -100 to +20 mV in 5 mV steps and following 0 mV test pulse (20 ms) with interval of 4s.

### Solutions

For sodium current recording, pipette solution contained (in mM) CsCl 130, NaCl 10, CaCl_2 _1, MgCl_2 _2, EGTA 10, HEPES 10, Tris-ATP 5 at pH 7.3 and osmolality 300mOsm. The external solution was composed of (in mM): NaCl 30, KCl 5, MgCl_2 _3, TEA-Cl 20, Choline-Cl 35, 4-AP 3, D-Mannitol 106, HEPES 10 at pH 7.4 and osmolality 300mOsm. 300nM TTX was added in the external solution to separate TTX-S current. Hypotonic external solutions were obtained by adjusting the concentration of D-Mannitol. The osmolality was measured using a vapor pressure osmometer (Model 3300, Advanced Instruments, Norwood, MA).

### Data analysis

Data were analyzed using pClamp (Axon Instruments) and SigmaPlot (SPSS Inc., Chicago, IL, USA) software. All data were presented as mean ± S.E.M. and the significance was indicated as P < 0.05 (*) and P < 0.01(**) tested by paired or unpaired Student's *t*-tests. The amplitude of sodium current was calculated as peak current. G-V curve and inactivation-voltage curve were fitted by Boltzmann functions, which G/G_max _= 1/(1 + exp (V_0.5 _- V_m_)/*k*) or I/I_max _= 1/(1 + exp (V_0.5 _- V_m_)/*k*), with V_0.5 _being membrane potential (V_m_) at which 50% of activation or inactivation was observed and *k *being the slope of the function. The dose-response curve was fitted by Hill equation, in which *I*_peak _= *I*_peakmax_/[1+(EC_50_/*C*)^*n*^], with *n *as the Hill coefficient, and EC_50 _as the concentration producing 50% increase.

### Chemicals

Cell culture materials were purchased from GIBCO (Life Technologies, Rockville, MD, USA). Others came from Sigma Chemical Company.

## Competing interests

The authors declare that they have no competing interests.

## Authors' contributions

LC conceived and designed the study. LL and CJL performed the experiments. Manuscript was written by LC and LgC. The final manuscript was read and approved by all authors.

## Supplementary Material

Additional file 1 **Table S1 Effect of second messenger systems on TTX-S current**. Click here for file

## References

[B1] Alessandri-Haber N Yeh JJ Boyd AE Parada CA Chen X Reichling DB Levine JD Hypotonicity induces TRPV4-mediated nociception in rat Neuron 2003 39 497 511 10.1016/S0896-6273(03)00462-8 12895423

[B2] Chen X Alessandri-Haber N Levine JD Marked attenuation of inflammatory mediator-induced C-fiber sensitization for mechanical and hypotonic stimuli in TRPV4^-/- ^mice Mol Pain 2007 3 31 37 10.1186/1744-8069-3-31 17967183 PMC2173885

[B3] Chen L Liu C Liu L Osmolality-induced tuning of action potentials in trigeminal ganglion neurons Neurosci Lett 2009 452 79 83 10.1016/j.neulet.2009.01.022 19444958 PMC2775522

[B4] Chahine M Ziane R Vijayaragavan K Okamura Y Regulation of Na v channels in sensory neurons Trends Pharmacol Sci 2005 26 496 502 10.1016/j.tips.2005.08.002 16125256

[B5] Wood JN Baker M Voltage-gated sodium channels Curr Opin Pharmacol 2001 1 17 21 10.1016/S1471-4892(01)00007-8 11712529

[B6] Chen L Liu C Liu L Cao X Changes in osmolality modulate voltage-gated sodium channels in trigeminal ganglion neurons Neurosci Res 2009 64 199 207 10.1016/j.neures.2009.02.012 19428701 PMC2684961

[B7] Black JA Cummins TR Plumpton C Chen YH Hormuzdiar W Clare JJ Waxman SG Upregulation of a silent sodium channel after peripheral, but not central, nerve injury in DRG neurons J Neurophysiol 1999 82 2776 2785 10561444 10.1152/jn.1999.82.5.2776

[B8] Black JA Liu S Tanaka M Cummins TR Waxman SG Changes in the expression of tetrodotoxin-sensitive sodium channels within dorsal root ganglia neurons in inflammatory pain Pain 2004 108 237 247 10.1016/j.pain.2003.12.035 15030943

[B9] Todaka H Taniguchi J Satoh J Mizuno A Suzuki M Warm temperature-sensitive transient receptor potential vanilloid 4 (TRPV4) plays an essential role in thermal hyperalgesia J Biol Chem 2004 279 35133 35138 10.1074/jbc.M406260200 15187078

[B10] Liedtke W TRPV channels' role in osmotransduction and mechanotransduction Handb Exp Pharmacol 2007 179 473 487 full_text 17217074 10.1007/978-3-540-34891-7_28

[B11] Liedtke W Molecular mechanisms of TRPV4-mediated neural signaling Ann N Y Acad Sci 2008 1144 42 52 10.1196/annals.1418.012 19076362

[B12] Levine JD Alessandri-Haber N TRP channels: targets for the relief of pain Biochim Biophys Acta 2007 1772 989 1003 17321113 10.1016/j.bbadis.2007.01.008

[B13] Alessandri-Haber N Joseph E Dina OA Liedtke W Levine JD TRPV4 mediates pain-related behavior induced by mild hypertonic stimuli in the presence of inflammatory mediator Pain 2005 118 70 79 10.1016/j.pain.2005.07.016 16213085

[B14] Alessandri-Haber N Yeh JJ Boyd AE Parada CA Chen X Reichling DB Levine JD Hypotonicity induces TRPV4-mediated nociception in rat Neuron 2003 39 497 511 10.1016/S0896-6273(03)00462-8 12895423

[B15] Baker MD Wood JN Involvement of Na^+ ^channels in pain pathways Trends Pharmacol Sci 2001 22 27 31 10.1016/S0165-6147(00)01585-6 11165669

[B16] Dib-Hajj SD Fjell J Cummins TR Zheng Z Fried K LaMotte R Black JA Waxman SG Plasticity of sodium channel expression in DRG neurons in the chronic constriction injury model of neuropathic pain Pain 1999 83 591 600 10.1016/S0304-3959(99)00169-4 10568868

[B17] Abdulla FA Smith PA Changes in Na^+ ^channel currents of rat dorsal root ganglion neurons following axotomy and axotomy-induced autotomy J Neurophysiol 2002 88 2518 2529 10.1152/jn.00913.2001 12424291

[B18] Sleeper AA Cummins TR Dib-Hajj SD Hormuzdiar W Tyrrell L Waxman SG Black JA Changes in expression of two tetrodotoxin resistant sodium channels and their currents in dorsal root ganglion neurons after sciatic nerve injury but not rhizotomy J Neurosci 2000 20 7279 7289 11007885 10.1523/JNEUROSCI.20-19-07279.2000 PMC6772759

[B19] Waxman SG Kocsis JD Black JA Type III sodium channel mRNA is expressed in embryonic but not adult spinal sensory neurons, and is reexpressed following axotomy J Neurophysiol 1994 72 466 470 7965028 10.1152/jn.1994.72.1.466 PMC2605356

[B20] Liu L Chen L Liedtke W Simon SA Changes in osmolality sensitize the response to capsaicin in trigeminal sensory neurons J Neurophysiol 2007 97 2001 2015 10.1152/jn.00887.2006 17353553

[B21] O'Neil RG Heller S The mechanosensitive nature of TRPV channels Pflugers Arch 2005 451 193 203 15909178 10.1007/s00424-005-1424-4

[B22] Grant AD Cottrell GS Amadesi S Trevisani M Nicoletti P Materazzi S Altier C Cenac N Zamponi GW Bautista-Cruz F Lopez CB Joseph EK Levine JD Liedtke W Vanner S Vergnolle N Geppetti P Bunnett NW Protease-activated receptor 2 sensitizes the transient receptor potential vanilloid 4 ion channel to cause mechanical hyperalgesia in mice J Physiol 2007 578 715 733 10.1113/jphysiol.2006.121111 17124270 PMC2151332

[B23] Chen L Liu C Liu L The modulation of voltage-gated potassium channels by anisotonicity in trigeminal ganglion neurons Neuroscience 2008 154 482 495 10.1016/j.neuroscience.2008.03.046 18456412 PMC2517136

[B24] Chen L Liu C Liu L Changes in osmolality modulate voltage-gated calcium channels in trigeminal ganglion neurons Brain Res 2008 1208 56 66 10.1016/j.brainres.2008.02.048 18378217 PMC2442870

[B25] Stamboulian S Choi JS Ahn HS Chang YW Tyrrell L Black JA Waxman SG Dib-Hajj SD ERK1/2 mitogen-activated protein kinase phosphorylates sodium channel Na(v)1.7 and alters its gating properties J Neurosci 2010 30 1637 1647 10.1523/JNEUROSCI.4872-09.2010 20130174 PMC6633991

[B26] Blair NT Bean BP Roles of tetrodotoxin (TTX)-sensitive Na^+ ^current, TTX-resistant Na^+ ^current, and Ca^2+ ^current in the action potentials of nociceptive sensory neurons J Neurosci 2002 22 10277 10290 12451128 10.1523/JNEUROSCI.22-23-10277.2002 PMC6758735

[B27] Scholz A Gruss M Vogel W Properties and functions of calcium-activated K^+ ^channels in small neurons of rat dorsal root ganglion studied in a thin slice preparation J Physiol 1998 513 55 69 10.1111/j.1469-7793.1998.055by.x 9782159 PMC2231273

