# Supplementary material for: Hypotonicity modulates tetrodotoxin-sensitive sodium current in trigeminal ganglion neurons
Source: Mol Pain. 2011 Apr 16;7:27. doi: 10.1186/1744-8069-7-27 (PMC3094255; doi:10.1186/1744-8069-7-27)
Supplement: Additional file 1 — Table S1 Effect of second messenger systems on TTX-S current. [file 1744-8069-7-27-S1.DOC]

**Table 1 Effect of second messenger systems on TTX-S current**

| Second messenger system | | TTX-S current (pA/pF) | | n |
| --- | --- | --- | --- | --- |
|  |  | 300mOsm | 300+angonist (antagonist) |  |
| PKA | 8-Br-cAMP (1 mM) | –160.47 ± 9.75 | -146.05±3.46* | 7 |
|  | H-89 (10 μM) | -169.51±6.86 | -195.35±7.14* | 7 |
|  | KT5720 (1 μM) | -161.11±8.14 | -180.32±9.11* | 7 |
| PKC | PMA (1 μM) | -168.09±7.26 | -152.60±8.31* | 7 |
|  | BIM (1 μM) | -168.95±7.90 | -184.48±8.01* | 8 |
|  | Staurosporine (1 μM) | -165.75±4.73 | -190.05±3.19* | 7 |

Paired t-test was used when comparing the effect of agonists or antagonists on TTX-S current in the isotonic solution (300mOsm). **P*<0.05
